# Mechanisms of Cisplatin Nephrotoxicity

**DOI:** 10.3390/toxins2112490

**Published:** 2010-10-26

**Authors:** Ronald P. Miller, Raghu K. Tadagavadi, Ganesan Ramesh, William Brian Reeves

**Affiliations:** Division of Nephrology, The Pennsylvania State University College of Medicine, 500 University Drive, Hershey, PA 17033, USA; Email: RMiller8@hmc.psu.edu (R.P.M.); rkt113@psu.edu (R.K.T.); GRamesh@hmc.psu.edu (G.R.)

**Keywords:** cisplatin, inflammation, kidney injury, nephrotoxicity, apoptosis, dendritic cells, cytokines, toll-like receptors

## Abstract

Cisplatin is a widely used and highly effective cancer chemotherapeutic agent. One of the limiting side effects of cisplatin use is nephrotoxicity. Research over the past 10 years has uncovered many of the cellular mechanisms which underlie cisplatin-induced renal cell death. It has also become apparent that inflammation provoked by injury to renal epithelial cells serves to amplify kidney injury and dysfunction *in vivo*. This review summarizes recent advances in our understanding of cisplatin nephrotoxicity and discusses how these advances might lead to more effective prevention.

## 1. Introduction

Cisplatin (*cis-* diamminedichloroplatinum(II), CDDP) is an antineoplastic drug used in the treatment of many solid-organ cancers, including those of the head, neck, lung, testis, ovary, and breast. While toxicities include ototoxicity, gastrotoxicity, myelosuppression, and allergic reactions [1,2], the main dose-limiting side effect of cisplatin is nephrotoxicity [3,4,5]. The nephrotoxicity of cisplatin has been recognized since its introduction over 25 years ago. Yet, in spite of intense efforts over the ensuing decades to find less toxic but equally effective alternatives, cisplatin continues to be widely prescribed. It remains as a standard component of treatment regimens for head and neck cancers [6], testicular cancer [7], small-cell [8] and non-small cell lung cancer [9], ovarian [10,11] and cervical cancer [12], bladder cancer [13] and others [14]. Cisplatin is available as a generic drug in the United States, making the tracking of sales and use difficult. However, a search of the ClinicalTrials.gov database returned 543 active treatment trials involving cisplatin as an indication of its ongoing wide clinical use. 

Cisplatin nephrotoxicity can present in a number of ways (Table 1). However, the most serious and one of the more common presentations is acute kidney injury (AKI) which occurs in 20–30% of patients. This review focuses on the mechanisms of cisplatin-induced acute kidney injury. We will briefly discuss the clinical features of cisplatin-induced AKI followed by a more detailed discussion of the responsible cellular mechanisms, with a particular emphasis on the role of inflammation in organ dysfunction. We will conclude with a consideration of mechanistically-targeted preventive measures. 

**Table 1:** Renal manifestations of cisplatin treatment.

Acute kidney injury (20–30%)	[15,16]
Hypomagnesemia (40–100%)	[17,18,19,20,21]
Fanconi-like syndrome	[22,23,24,25,26]
Distal renal tubular acidosis	[27]
Hypocalcemia	[28,29]
Renal salt wasting	[22,30,31,32,33,34,35,36]
Renal concentrating defect	[22,34,37,38,39,40]
Hyperuricemia	[41]
Transient proteinuria	[42]
Erythropoietin deficiency	[43]
Thrombotic microangiopathy	[44]
Chronic renal failure	[15,45,46]

## 2. Clinical Characteristics of Cisplatin Nephrotoxicity

Cisplatin was first shown to inhibit cell division in 1965 [47]. By 1969, cisplatin was found to have anti-tumor effects in animal models [48]. The first report of nephrotoxicity in animal studies was in 1971 [49], which demonstrated histopathologic changes of acute tubular necrosis along with azotemia. Early clinical use of cisplatin saw dose-related cisplatin-induced acute renal failure in 14 to 100% of patients, with the incidence varying with the cumulative dose [15,16]. The incidence of renal insufficiency in more recent experience using saline hydration and diuresis, is in the range of 20–30% of patients [50]. Typically, the onset of renal insufficiency begins several days after the dose of cisplatin, as revealed by increases in the serum creatinine and blood urea nitrogen concentrations. The urine output is usually preserved (non-oliguric) and the urine may contain glucose and small amounts of protein, indicative of proximal tubular dysfunction. Hypomagnesemia is also common, particularly after repeated doses of cisplatin, even in the absence of a fall in the glomerular filtration rate. Recovery of renal function usually occurs over a period of 2–4 weeks, though more protracted courses, as well as lack of recovery are reported. Progressive and permanent nephrotoxicity can result with successive treatment courses despite preventative measures [51,52]. 

A number of risk factors for cisplatin nephrotoxicity have been identified (Table 2). Nephrotoxicity increases with the dose and frequency of administration and cumulative dose of cisplatin [15]. High peak plasma free platinum concentration has been correlated with nephrotoxicity [53], and one study has suggested glomerular filtration rate and plasma magnesium concentrations decreased after cisplatin doses higher than 50 mg/m^2^ body surface area, but were unchanged if the dose was below 20 mg/m^2^ [50]. Other patient variables have been found to associate with increased risk of nephrotoxicity, including female sex, older age, smoking, and hypoalbuminemia [54,55]. In general, pre-existing renal dysfunction increases the risk for AKI. In the specific case of cisplatin, however, there are limited data on the incidence of nephrotoxicity in populations with chronic kidney disease since many trials exclude patients with renal insufficiency [56]. Diabetes decreases the risk of cisplatin nephrotoxicity in animal models [57], but clinical studies have not found any impact of diabetes on nephrotoxicity in humans [58,59]. Patients with a certain polymorphism in the OCT2 gene, which regulates platinum transport into kidney cells, may also be at lower risk of nephrotoxicity [60,61].

**Table 2:** Risk factors for cisplatin nephrotoxicity.

**Increased risk**
Dose
Frequency
Cumulative dose
Older age
Female sex
Smoking
Hypoalbuminemia
Pre-existing renal insufficiency (limited data in humans)
**Decreased risk**
Diabetes (uncertain in humans)
OCT2 polymorphisms

## 3. Mechanisms of Cisplatin Nephrotoxicity

### 3.1. Accumulation of Cisplatin in Kidney Cells

Cisplatin is cleared by the kidney by both glomerular filtration and tubular secretion [62]. Cisplatin concentrations within the kidney exceed those in blood suggesting an active accumulation of drug by renal parenchymal cells. Previous studies using kidney slices [63], cultured renal epithelial cells [64] and isolated perfused proximal tubule segments [65] have provided evidence for basolateral-to-apical transport of cisplatin. Studies in recent years have identified two different membrane transporters capable of transporting cisplatin into cells: Ctr1 and OCT2. Ctr1 is a copper transporter which was also shown to mediate cisplatin uptake into mammalian cells [66], including ovarian cancer cells [67]. Ctr1 is highly expressed in adult kidney and the protein localizes to the basolateral membrane of the proximal tubule [68]. Downregulation of Ctr1 expression in kidney cells *in vitro* decreased both cisplatin uptake and cytotoxicity, suggesting that Ctr1 is an important cisplatin uptake mechanism in these cells [68]. The role of Ctr1 in cisplatin nephrotoxicity *in vivo* has not been examined. In addition, the organic cation transporter OCT2 (SLC22A2) transports cisplatin [69,70,71,72]. Cisplatin was shown to inhibit the uptake of other OCT2 substrates, consistent with the view that these substrates share a common transport pathway. Likewise, cimetidine, an OCT2 substrate, reduced cisplatin uptake and cytotoxicity *in vitro* [68,69,70] and cisplatin nephrotoxicity *in vivo* [61]. Two recent observations point to an important role for OCT2 in mediating renal cisplatin uptake and toxicity. First, knockout of the OCT2 gene significantly reduced urinary cisplatin excretion [60] and nephrotoxicity [60,61]. Second, a nonsynonymous single-nucleotide polymorphism (SNP) in the OCT2 gene (rs316019) was associated with reduced cisplatin-induced nephrotoxicity in patients [60,61]. The relevance of these findings to the possible prevention of cisplatin nephrotoxicity is discussed later. 

### 3.2. Biotransformation of Cisplatin in the Kidney

Studies in rats and mice indicate that cisplatin undergoes metabolic activation in the kidney to a more potent toxin. This process begins with the formation of glutathione conjugates in the circulation, perhaps mediated by glutathione-S-transferase [73,74]. As the glutathione-conjugates pass through the kidney, they are cleaved to cysteinyl-glycine-conjugates by gamma glutamyl transpeptidase (GGT) expressed on the surface of the proximal tubule cells [75,76]. The cysteinyl-glycine-conjugates are further metabolized to cysteine-conjugates by aminodipeptidases, also expressed on the surface of the proximal tubule cells [75]. The cysteine-conjugates are transported into the proximal tubule cells, where they are further metabolized by cysteine-S-conjugate beta-lyase to highly reactive thiols [75,76,77]. 

### 3.3. Cellular Targets of Cisplatin

**Figure 1:**
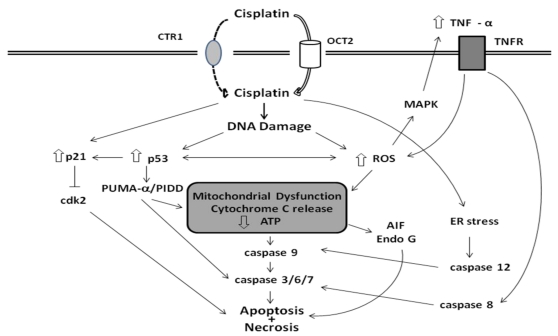
Pathways of cisplatin-induced epithelial cell death. Cisplatin enters renal epithelial cells via the OCT2 and, to a lesser extent, Ctr1 transporters. Cisplatin causes damage to nuclear and mitochondrial DNA and production of reactive oxygen species (ROS) which lead to activation of both mitochondrial and non-mitochondrial pathways of apoptosis and necrosis.

Platinum compounds are believed to mediate their cytotoxic effects through their interaction with DNA (Figure 1). In an aqueous environment, the chloride ligands of cisplatin are replaced by water molecules generating a positively charged electrophile. This electrophile reacts with nucleophilic sites on intracellular macromolecules to form DNA, RNA, and protein adducts [78]. Cisplatin binds to DNA leading to the formation of inter- and intrastrand cross-links, thereby arresting DNA synthesis and replication in rapidly proliferating cells [79]. The finding that cells deficient in DNA repair are more sensitive to cisplatin-induced cell death supports the concept that cisplatin mediates its anti-tumor effects through DNA damage. However, the primacy of nuclear DNA damage as the cause of cisplatin‑induced cell death has been challenged. In fact, only a small amount of cellular platinum (<1%) is bound to nuclear DNA and there is a poor correlation between the sensitivity of cells to cisplatin-induced cell death and the extent of DNA platination [80]. Moreover, Mandic *et al.* [81] used enucleated cells to demonstrate that cisplatin-induced apoptotic signaling occurs independently of nuclear DNA damage. 

Several lines of evidence suggest that mitochondrial DNA, or other mitochondrial targets, are perhaps more important than nuclear DNA damage in mediating cisplatin-induced cell death [82]. Cisplatin is hydrolyzed to generate a positively charged metabolite which preferentially accumulates within the negatively charged mitochondria. Thus, the sensitivity of cells to cisplatin appears to correlate with both the density of mitochondria [83] and the mitochondrial membrane potential [84]. This observation may explain the particular sensitivity of the renal proximal tubule to cisplatin toxicity, as this segment exhibits one of the highest densities of mitochondria in the kidney [85]. A comparison of cisplatin-sensitive and cisplatin-resistant ovarian cancer cells revealed a lower mitochrondrial membrane potential as well as less damage to mitochondrial DNA in the latter [84]. Moreover, depletion of mitochondrial DNA by growth of cells in ethidium bromide rendered cells highly resistant to cisplatin [83]. Finally, mitochondrial DNA may be more susceptible than nuclear DNA to cisplatin-induced damage, due to less efficient DNA repair mechanisms [86]. Taken together, these observations point to mitochondrial DNA as an important target in cisplatin toxicity. 

Mitochondrial energetics are also disrupted by cisplatin and may contribute to nephrotoxicity. Fatty acids are the major source of energy for the proximal tubule, the primary site of cisplatin kidney injury. Cisplatin inhibits fatty acid oxidation in mouse kidney and in proximal tubule cells in culture through a reduction in PPAR-α mediated expression of genes involved in cellular fatty acid utilization [87,88]. Agonists of PPAR-α reduce cisplatin nephrotoxicity *in vivo* [87,89]. Cisplatin also affects mitochondrial respiratory complexes and function. Exposure of cultured proximal tubule cells to cisplatin *in vitro* inhibited mitochondrial complexes I to IV of the respiratory chain and, as a result, decreased intracellular ATP levels [90]. Cisplatin treatment *in vivo* also resulted in mitochondrial dysfunction as evidenced by a decline in membrane electrochemical potential, a substantial decrease in mitochondrial calcium uptake and depletion of mitochondrial antioxidant defense systems [90,91]. 

### 3.4. Apoptotic Pathways of Cisplatin Cytotoxicity

The mechanisms of cisplatin-induced nephrotoxicity are complex and involve multiple pathways and molecules (Figure 1) [92,93]. The cellular pathways of cisplatin injury to kidney cells have been examined primarily *in vitro* using freshly isolated or cultured renal tubular epithelial cells. *In vitro*, low concentrations of cisplatin preferentially result in apoptotic cell death while at higher concentrations necrosis ensues [94,95]. *In vivo* administration of nephrotoxic doses of cisplatin produces a large increase in both necrosis and apoptosis in the kidney [96,97,98]. Several apoptotic pathways have been implicated in cisplatin-induced renal epithelial cell death, including the extrinsic pathway activated through death receptors, such as TNF receptors or Fas, the intrinsic mitochondrial pathway and the endoplasmic reticulum stress pathway. Evidence that death receptor pathways may be activated by cisplatin include observations that TNFR1 and Fas-deficient renal epithelial cells are resistant to cisplatin-induced cell death [99], that cisplatin increases the activity of caspase 8 [100] and that inhibition of caspase 8 reduces cisplatin-induced cell death *in vitro* [101]. As will be discussed below, TNF-α stimulates an inflammatory response *in vivo* which exacerbates cisplatin nephrotoxicity [99,102,103]. The relative importance of TNF-α directly engaging TNF receptors on renal epithelial cells to induce apoptosis *versus* its role in promoting inflammation is not clear. Additional studies using tissue-specific deletions of TNF receptors will be needed to address this issue. In contrast, there is a large body of evidence indicating that cisplatin activates the intrinsic mitochondrial pathway of apoptosis. Thus, exposure of renal epithelial cells to cisplatin results in the translocation of Bax to mitochondria, activation of caspase 2, release of cytochome c, AIF, SMAC/Diablo, Omi/HtrA2 and endonuclease G from mitochondria and activation of caspase 9 [95,104,105,106,107,108,109,110,111]. Caspases are a family of cell death proteases that play an essential role in the execution phase of apoptosis in cisplatin induced renal tubular epithelial cell death *in vitro* and *in vivo* [109,112,113,114]. Activation of caspases 3, 8 and 9 occur as early as 12 hours after cisplatin treatment of renal epithelial cells *in vitro* [113] and inhibition of caspase activity suppresses cisplatin induced cell death [109,113,114,115]. Both p53 dependent expression of caspases 6 and 7 [114] and p53‑independent activation of caspases through Bax/Bak mediated release of cytochrome C [109] contribute to cisplatin induced tubular epithelial cell death. The ER stress pathway involves activation of caspase 12 and Ca^2+^ dependent phospholipase A_2_ and pharmacological inhibition of these enzymes reduces cisplatin-induced apoptosis [116,117,118]. Finally, autophagy has recently been shown to participate in cisplatin-induced cell injury. Autophagy is a cellular process of degradation of damaged organelles, protein aggregates and other macromolecules in the cytoplasm. Treatment of renal epithelial cells with cisplatin causes the rapid expression of autophagic proteins and the formation of autophagosomes [119,120,121]. Inhibition of autophagy resulted in accelerated apoptosis indicating a protective role for autophagy in the cellular response to cisplatin [119,121].

Cell cycle regulators also play an important role in tubular cell damage [122,123]. Shortly after AKI many normally quiescent kidney cells enter the cell cycle. Control of the cell cycle is determined by the sequential activation and inhibition of the cyclin-dependent kinases (e.g., cdk2). p21, a cyclin dependent kinase inhibitor, is upregulated in kidney after cisplatin treatment and plays a protective role against toxicity. Thus, overexpression of p21 inhibits cisplatin-induced apoptosis *in vitro* while mice lacking the p21 gene are more sensitive to cisplatin nephrotoxicity *in vivo* [97,123,124]. The protective effects of p21 are due to its inhibition of cdk2, a cell cycle-associated kinase primarily active during late G1 through S phases [124,125]. Presumably, by inhibiting progression through the cell cycle, p21 allows time for cells to repair cisplatin-induced DNA damage.

p53 has gained attention as a major mediator of cisplatin-induced cell death. The p53 tumor suppressor induces cell cycle arrest or apoptosis in response to DNA damage, oncogene activation, and hypoxia [126]. Cisplatin treatment activates p53 in kidney *in vivo* [127] and renal epithelial cells *in vitro* [100,115,128]. Moreover, pharmacologic or genetic inhibition of p53 transcriptional activity reduced cisplatin-induced caspase activation and apoptosis *in vitro* [100,115,128], and cisplatin-induced apoptosis and renal injury *in vivo* [127,129]. Two targets of p53 transcriptional regulation, p53 up‑regulated modulator of apoptosis-alpha (PUMA-α) and p53-induced protein with a death domain (PIDD), may mediate p53 actions in cisplatin cell death. PUMA-α is a proapoptotic Bcl-2 family protein which is induced by cisplatin in a p53-dependent manner [107]. Activation of p53 by cisplatin also induces PIDD, which then activates caspase 2, leading to mitochondrial release of AIF [100]. p53 may translocate to mitochondria during cell stress where it has certain non-transcriptional actions, such as maintenance of mitochondrial DNA copy number and production of reactive oxygen species [130,131]. However, the specific role of mitochondrial p53 in cisplatin nephrotoxicity is not known. The mechanism of p53 activation by cisplatin may involve DNA damage and oxidative stress [132,133]. DNA fragmentation in response to cisplatin is mediated by DNAse I and endonuclease G [134,135]. DNase I may introduce initial ssDNA breaks after being passively translocated to nuclei. After the initial DNA damage produced by DNase I or cisplatin, DNA becomes more susceptible to EndoG digestion [135]. 

Histone acetylation may be a target of cisplatin injury in kidney cells. Histone deacetylase inhibitors are being developed as anti-cancer agents. At high concentrations, these agents, such as suberoylanilide hydroxamic acid (SAHA) and Trichostatin A, induce apoptosis in renal epithelial cells [136]. However, in lower doses they appear to be protective against cisplatin-induced cell death *in vitro* [137,138]. Histone deacetylase inhibitors might, however, exacerbate the inflammatory response seen in cisplatin nephrotoxicity. For example, histone deacetylases, in conjunction with the transcriptional repressor, activating transcription factor 3 (ATF3), inhibited the transcription of inflammation-related genes during renal ischemic injury [139]. The effects of histone deacetylase inhibitors on cisplatin nephrotoxicity *in vivo* have not been reported. 

Cellular stress induced by cisplatin also activates MAPK pathways (ERK, p38 and JNK). Inhibition of p38 MAPK, ERK or JNK with specific pharmacologic or genetic inhibitors reduced apoptosis, caspase activation, inflammation and renal injury [140,141,142,143,144]. Cisplatin-induced production of reactive oxygen species has also been implicated in its direct cellular toxicity [96,145,146]. In this regard, cisplatin injury can be ameliorated by free radical scavengers [147,148], iron chelators [145], superoxide dismutase [146], catalase [149], selenium and Vitamin E [150] and heme oxygenase-1 induction [96]. 

In summary, cisplatin-induced renal cell death involves multiple pathways including oxidant stress, activation of intrinsic and extrinsic apoptotic cascades and endonucleases (Figure 1). Unfortunately, many of these same pathways contribute to the cytotoxic actions of cisplatin on tumor cells. Therefore, strategies intended to reduce cisplatin renal injury may have the unintended consequence of reducing the anti-tumor actions of cisplatin. The design of preventive strategies must carefully consider this risk. 

## 4. Inflammation in Cisplatin Nephrotoxicity

**Figure 2:**
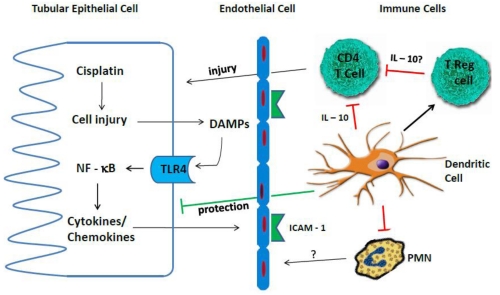
Immune mechanisms of cisplatin nephrotoxicity. Cisplatin-induced injury to renal epithelial cells causes release of DAMPs, which activate TLR4. Activation of TLR4 results in the production of a variety of chemokines and cytokines, including TNF-α. These chemokines and cytokines upregulate adhesion molecules and attract inflammatory cells, such as neutrophils and T cells, into the region of injury. Tissue resident dendritic cells act to reduce kidney injury, at least in part by producing the anti-inflammatory cytokine IL-10. Treg cells also reduce kidney injury although the mechanism is still unknown. Dendritic cells may enhance the number or activity of Treg cells, though this has not been demonstrated.

There is a growing recognition of the importance of inflammation, in addition to direct cellular toxicity, in the pathogenesis of cisplatin nephrotoxicity. Over the past 10 years, a number of the mediators of inflammatory renal injury have been identified (Figure 2).

### 4.1. Cytokines

TNF-α is the prototypical inflammatory cytokine and plays a central role in many infectious and inflammatory diseases. Anti-TNF therapies are widely used for certain inflammatory diseases such as psoriasis, rheumatoid arthritis and inflammatory bowel disease. An increase in renal expression of TNF-α was demonstrated in a mouse model of cisplatin nephrotoxicity by Kelly *et al.* [151] and Deng *et al.* [152]. To address the functional relevance of TNF-α in the pathogenesis of cisplatin-induced acute renal failure, renal function and renal histology were examined in mice treated with cisplatin in the presence or absence of TNF-α inhibitors and also in TNF-α knockout mice [102]. Treatment with TNF-α inhibitors reduced cisplatin-induced renal dysfunction and also reduced histologic evidence of injury. TNF knockout mice also sustained less renal injury than wild type mice and had markedly higher survival rates following cisplatin injection [102]. These results, which have been confirmed by a number of laboratories [99,153], establish an important role for TNF-α in the pathogenesis of cisplatin nephrotoxicity. 

TNF-α can be produced by a variety of immune and non-immune cells. To determine the source of TNF which resulted in cisplatin nephrotoxicity, Zhang *et al.* [154] created chimeric mice in which the bone marrow was ablated and replaced with donor bone marrow cells from either wild-type or from TNF-α knockout mice. Chimeras with kidneys of wild-type animals developed significant renal failure after cisplatin treatment regardless of the immune cell source. Chimeras with kidneys of TNF-α knockout mice showed significantly less renal dysfunction, renal histologic injury, and urine and serum TNF-α levels; again regardless of the immune cell source. These results indicate that a substantial portion of circulating and urinary TNF-α is derived from non-immune cells, probably renal epithelial cells themselves, after cisplatin administration. The production of TNF-α after cisplatin administration is highly dependent upon reactive oxygen species, NFκB activation and activation of p38 MAPK. DMTU, a hydroxyl radical scavenger, salicylate (inhibitor of IKK) and p38 inhibitors, reduced both TNF-α production and nephrotoxicity in mice treated with cisplatin [98,142].

The biological activities of TNF-a are mediated by two functionally distinct receptors, TNFR1 (p55) and TNFR2 (p75). Many of the cytotoxic and proinflammatory actions of TNF-a are mediated by TNFR1 [155,156]. However, studies in mice deficient in either TNFR1 or TNFR2 revealed that the nephrotoxic effects of cisplatin, at least those mediated by TNF-α, are signaled through TNFR2 rather than TNFR1 [103]. 

The expression of a number of inflammatory cytokines and chemokines is increased in the kidney after cisplatin injury. However, evidence for a functional role for many of these cytokines is lacking. For example, Edelstein’s group [157,158] determined that the expression of IL-1β, IL-18, CX3CL1 and IL-6 were increased in cisplatin nephrotoxicity. In addition, deletion of caspase 1, which is responsible for the formation of active IL-1β and IL-18, reduced cisplatin kidney injury and neutrophil infiltration *in vivo* [159]. However, inhibition or genetic deletion of these cytokines did not reduce cisplatin nephrotoxicity, leaving the mechanism by which caspase 1 contributes to cisplatin injury uncertain. Likewise, IFN-γ expression is increased in cisplatin nephrotoxicity, but neutralizing antibodies to IFN-γ provided no protection against renal injury [160]. It is interesting that many of these cytokines are upregulated in a TNF-dependent fashion [102]. It may be that these individual downstream cytokines each have minor roles in cisplatin nephrotoxicity which are difficult to demonstrate experimentally while inhibition of the upstream TNF-α produces more dramatic effects due to the cumulative actions of multiple downstream cytokines. 

Cytokines can also exert anti-inflammatory actions. IL-10 is an anti-inflammatory cytokine that suppresses the activation of leukocytes and the production of proinflammatory cytokines and chemokines [161]. Deng *et al.* [152] demonstrated that the injection of exogenous IL-10 inhibits the upregulation of TNF-α and ICAM-1 expression and the influx of neutrophils into the kidney in response to cisplatin. We have recently determined that endogenous production of IL-10 is an important defense mechanism against cisplatin nephrotoxicity. IL-10 deficient mice were more susceptible to cisplatin nephrotoxicity and exhibited greater influx of neutrophils and higher expression of proinflammatory cytokines than wild type mice [160]. At least part of this effect was due to IL-10 production by dendritic cells. 

In addition to their roles in the pathogenesis of cisplatin nephrotoxicity, cytokines may also have diagnostic value for this disorder. The levels of several pro-inflammatory cytokines, including TNF-α, IL-6, IL-2, IP-10, MCP-1 and KC, are increased in the urine of cisplatin-treated mice [162]. We have determined that elevations in IP-10, KC and G-CSF are detectable in the urine as early as six hours after cisplatin treatment, long before the serum creatinine or urea nitrogen levels increase [163]. Likewise, increases in urinary KC, IL-2, MCP-1, GM-CSF and IL-8 levels were noted after three days of low-dose cisplatin treatment in dogs [164]. Measurement of urinary cytokines may allow detection of early cisplatin toxicity and may be useful endpoints in trials of preventive strategies. 

### 4.2. TLR Receptors

Toll like receptors (TLRs) are a family of pattern recognition receptors which detect components, such as RNA, DNA or proteins, of foreign organisms [165]. They play a pivotal role in host immunity to infection by sensing the invasion of organisms and initiating both innate and adaptive immune responses. In addition to detecting foreign invaders, TLRs also detect and respond to certain endogenous molecules, termed “alarmins” or damage-associated molecular pattern molecules (DAMPs), associated with tissue injury [166]. LPS, an agonist of TLR4, can induce AKI when administered at high doses [167,168]. More recent studies determined that low doses of LPS, insufficient to cause renal injury, can exacerbate kidney injury induced by other insults, including cisplatin [169,170,171]. This synergistic effect of LPS and cisplatin was dependent on both TLR4 and TNF-α and raised the possibility that TLR4 was involved in the response to both agents [171]. This view was confirmed by Zhang *et al.* who demonstrated that cisplatin-treated wild-type mice incurred significantly more renal dysfunction, histologic damage, and leukocyte infiltration in the kidney than similarly treated mice with a targeted deletion of TLR4 [162]. Levels of cytokines, including TNF-α, in serum, kidney, and urine were also significantly higher in cisplatin-treated wild-type mice compared with cisplatin-treated TLR4 KO mice. Activation of p38, which is critical for cisplatin-induced TNF-α production [142], was significantly blunted in TLR4 KO mice. Using bone marrow chimeric mice, they also determined that renal parenchymal TLR4, rather than myeloid TLR4, mediated the nephrotoxic effects of cisplatin. Based on these findings, TLR4 appears to be a sensor for cisplatin‑induced epithelial injury. Once activated, TLR4 on renal parenchymal cells may activate p38 MAPK pathways, leading to increased production of inflammatory cytokines, such as TNF-α and subsequent kidney injury. The ligand responsible for TLR4 activation in cisplatin nephrotoxicity is unknown. The nuclear protein HMGB1 has been shown to activate TLR4 in various pathologic settings [166,172]. However, cisplatin actually increases binding of HMBG1 to DNA and inhibits its release from cells [173]. Other putative DAMPs include gp96, HSP60, HSP70 and β-defensin-2 [162]. The roles of these other DAMPs in cisplatin nephrotoxicity have not been reported. The possible clinical implications of TLR4 signaling in terms of preventing cisplatin nephrotoxicity will be discussed later. 

Other TLR receptors, such as TLR2 and TLR9 have been implicated in tissue injury [174,175,176,177]. The possibility that TLR9 may be involved in cisplatin nephrotoxicity is particularly intriguing given recent evidence that TLR9 responds to mitochrondrial DAMPs [178] and the previously discussed cisplatin-induced injury to mitochondria. However, in preliminary studies from our laboratory TLR9 deficient mice were not protected from cisplatin renal injury [179]. The impact of TLR2 on cisplatin nephrotoxicity has not been reported.

### 4.3. Immune Cells

#### 4.3.1. Neutrophils

Cisplatin administration causes an increase in kidney neutrophil content [102,151,157,162,180]. Moreover, maneuvers which decrease cisplatin nephrotoxicity, such as inhibition of TNF-α or TLR4 signaling [98,102,103,162], inhibition of ICAM-1 [151] or administration of IL-10 [152], are associated with a decrease in renal neutrophil content. However, Faubel *et al.* [157] demonstrated that depletion of neutrophils using an anti-neutrophil antibody had no effect on cisplatin-induced renal dysfunction or tubular necrosis even though renal neutrophil infiltration was effectively abolished. These results suggest that infiltrating neutrophils are not essential for cisplatin-induced renal injury and may be a reflection of the severity of injury rather than its cause. 

#### 4.3.2. Macrophages

Macrophages have been implicated in the pathogenesis of ischemic AKI [181,182]. However, their role in cisplatin nephrotoxicity is uncertain. Treatment of peritoneal macrophages *in vitro* with cisplatin induces a pro-inflammatory phenotype characterized by increased production of nitric oxide and pro-inflammatory cytokines and activation of NFκB [183,184]. These attributes could contribute to an inflammatory response in the kidney. Lu *et al.* reported a 2-fold rise in kidney macrophages after cisplatin administration [158]. They also determined that the influx of macrophages was critically determined by CX3CL1 (fractalkine), a potent chemoattractant for macrophages. Nonetheless, inhibition of macrophage infiltration using either a CX3CL1 antibody or CX3CL1 deficient mice, or depletion of macrophages using liposomal clodronate, did not prevent cisplatin-induced renal dysfunction [158]. The lack of effect of CX3CL1 inhibition and macrophage depletion in cisplatin kidney injury, contrasts with the beneficial effect of these maneuvers in ischemic renal injury [181,182] and points to insult-specific pathways of AKI. 

#### 4.3.3. T Cells

T cells have been shown to contribute to ischemic AKI [185]. To evaluate the possible role of T cells in cisplatin nephrotoxicity, Liu *et al.* [186] administered cisplatin to T cell deficient mice. The T cell deficient mice sustained less renal dysfunction and tubular injury and had better survival than the T cell replete mice. It was determined that the harmful effects of T cells were mediated by CD4 T cells and to a lesser extent CD8 T cells. 

#### 4.3.4. Treg Cells

Treg cells are a class of CD4 T cells (CD4^+^CD25^+^FoxP3^+^) which suppress effector and cytotoxic T lymphocyte responses. They also downmodulate the function and/or proliferation of other immune cells, such as macrophages, dendritic cells, B cells, NK cells and neutrophils [187]. Recent work from the Rabb [188] and Okusa [189] laboratories has demonstrated that Treg cells reduce the severity of ischemic AKI and speed its recovery. Lee *et al.* [190] recently examined the role of Tregs in cisplatin nephrotoxicity. When CD4^+^CD25^+^ T cells were adoptively transferred to T cell deficient (*nu/nu*) mice, cisplatin-induced renal dysfunction and mortality were reduced. In contrast, the transfer of CD4^+^CD25^−^ cells did not improve renal function. In addition, transfer of CD4^+^CD25^+^ Treg cells to normal mice reduced cisplatin-induced renal injury while depletion of endogenous Treg cells with a CD25 antibody exacerbated injury. These results indicate that endogenous Treg cells play a protective role against cisplatin-induced kidney injury. The mechanism whereby Treg cells reduce cisplatin nephrotoxicity remains to be determined. However, since Treg cells were protective in T cell deficient mice, at least part of the protection was independent of effects on other T cells. 

#### 4.3.5. Dendritic Cells

Dendritic cells are sentinels of the immune system and under steady state conditions induce tolerance by various mechanisms including production of TGF-β [191] or IL-10 [192] and induction of Treg cells via the ICOS-ICOS-ligand pathway [193]. In response to pathogens or products of tissue injury, dendritic cells mature and initiate immunity or inflammatory diseases [194,195]. In the kidney, dendritic cells form an extensive network throughout the tubulointerstitial compartment [182,196]. Dendritic cells had been shown to produce TNF-α during ischemic renal injury [197] and were presumed to be pathogenic in this disorder. CD11c-DTR transgenic mice express the simian diphtheria toxin receptor in dendritic cells driven by the promoter for CD11c, a dendritic cell-specific cell surface marker [194]. Injection of diphtheria toxin to these mice results in depletion of dendritic cells. Using this system, Tadagavadi and Reeves [180] examined the role of dendritic cells in cisplatin nephrotoxicity. Mice depleted of dendritic cells before or coincident with cisplatin treatment but not at later stages, experienced more severe renal dysfunction, tubular injury, neutrophil infiltration and greater mortality than nondepleted mice. Studies involving mixed bone marrow chimeras demonstrated that the worsening of cisplatin nephrotoxicity in dendritic cell depleted mice was not a result of the dying or dead dendritic cells themselves. After cisplatin treatment, expression of MHC class II decreased and expression of inducible co-stimulator ligand increased on renal dendritic cells. These results demonstrated that resident dendritic cells reduce cisplatin nephrotoxicity and its associated inflammation. Subsequent studies determined that the production of the anti-inflammatory cytokine IL-10 by dendritic cells was responsible for a portion of the protective effects of dendritic cells [160]. It remains to be determined if some of the protective effects of dendritic cells are mediated via Treg cells. 

## 5. Prevention of Cisplatin Nephrotoxicity

Volume expansion with sodium chloride has been the primary means to reduce cisplatin nephrotoxicity [198]. Although many hydration regimens include the use of either mannitol or furosemide, there is no good evidence that diuretics provide any added benefit [199,200]. In fact, one comparative trial found greater nephrotoxicity in patients who received saline plus mannitol compared with saline alone [51]. Hypertonic (3%) saline has also been advocated [17]. However, subsequent studies showed decreases in GFR despite the use of 3% saline [201,202]. Recently published clinical guidelines recommend prehydration with 0.9% saline and avoidance of diuretics [40]. 

Amifostine (2-(3-aminopropylamino)ethylsulfanyl phosphonic acid) is approved by the U.S. Food and Drug Administration for use in reducing cumulative nephrotoxicity of repeated cisplatin dosing in patients with advanced ovarian cancer [203]. Amifostine may derive its protective effects by providing a thiol group to normal *versus* malignant cells [204,205]. Data are limited regarding the use in amifostine in tumors other than ovarian carcinoma [203].

Unfortunately, even with aggressive hydration, renal toxicity still occurs. This has encouraged the development of more effective preventive strategies. These strategies can be organized according to putative mechanisms (Table 3). In each case, it is important to consider how the preventive strategy might affect the desired anti-tumor activity of cisplatin. Renal toxicity from cisplatin derives from the uptake and activation of platinum within the proximal tubule cell. Therefore, maneuvers which differentially reduce cisplatin uptake, or activation by the kidney relative to tumor cells, should reduce nephrotoxicity without impairing anti-tumor responses. In this regard, certain formulations of micellar cisplatin have reduced kidney excretion but good tumor penetration [206,207]. OCT2 mediates cisplatin uptake into kidney cells, but not into tumor cells. Cimetidine, an OCT2 substrate, reduced cisplatin nephrotoxicity in mice [208]. In a small trial in humans, the combination of cimetidine and verapamil preserved renal function during cisplatin treatment [209]. Additional studies using cimetidine, or other OCT2 substrates such as metformin, are warranted. 

**Table 3:** Experimental strategies to prevent cisplatin nephrotoxicity.

**Reduced renal cisplatin accumulation or activation**	
OCT2 inhibitors, e.g., cimetidine or metformin	[61,208]
Ctr1 inhibitors, e.g., copper	[68]
Micellar/liposomal cisplatin	[206,207]
Gamma-glutamyl transpeptidase inhibitors	[76,210]
Glutathione transferase inhibitors	[74]
**Anti-oxidants**	
Amifostine	[203]
BNP7787	[211]
*N*-acetyl cysteine	[212]
Superoxide dismutase	[23,146]
Catalase	[149]
Selenium and Vitamin E	[150]
Heme oxygenase-1 induction	[96]
Iron chelators, e.g., Desferoximine	[145]
Allopurinol plus ebselen	[213]
Milk thistle extract (silymarin)	[214]
Cannabidiol	[215]
Lycopene	[216]
**Anti-apoptosis**	
p53 inhibitors, e.g., pifithrin	[100,115,127,128,129]
HDAC inhibitors	[137,138]
Caspase inhibitors	[113]
p21agonists/CDK2 inhibitors	[123,124]
**Anti-inflammation**	
TNF-α antagonists	[102]
TLR4 antagonists	[162]
p38 inhibitors	[142]
JNK inhibitors	[141]
Salicylates	[98]
PPAR-α ligands, e.g., fibrates	[217]
PPAR-γ ligands, e.g. rosiglitazone	[218]
Alpha lipoic acid	[219]
IL-10	[152]

Many agents have been reported that interrupt the cell death machinery in cisplatin-treated kidney cells (Table 3). Unfortunately, many of these same pathways are responsible for the cytotoxic actions of cisplatin in cancer cells. One potential exception are histone deacetylase inhibitors. These agents are in clinical development for cancer treatment but also appear to reduce cisplatin cytotoxicity *in vitro* [137,138]. The effects of these agents on cisplatin nephrotoxicity *in vivo* need to be explored. 

Inflammation contributes to cisplatin nephrotoxicity *in vivo*. A number of anti-inflammatory substances reduce cisplatin nephrotoxicity in animal models (Table 3). For the most part, the effects of these agents on tumor responses to cisplatin have not been examined. Our laboratory has shown that inhibitors of TNF-α reduce cisplatin nephrotoxicity [102]. Although TNF-α was named for its ability to induce hemorrhagic necrosis of tumors [220], there is increasing evidence that TNF-α is produced by cancer cells and acts as an endogenous tumor promoter [221,222,223]. These observations raise the possibility that anti-TNF agents might reduce cisplatin nephrotoxicity without reducing, and perhaps even enhancing, tumor responses. We have also demonstrated a role for TLR4 in cisplatin nephrotoxicity [162]. Relatively little is known about the role of TLR4 in tumor growth or response to chemotherapy. The literature includes examples of TLR4 having both pro-tumor and anti-tumor activities [224]. An interesting recent study found that the release of HMGB1, a TLR4 agonist, from dying tumor cells stimulated dendritic cells to initiate an adjuvant anti-tumor immune response in a TLR4 dependent fashion [225]. Accordingly, tumor-bearing TLR4 deficient mice had reduced responses to chemotherapy and radiation therapy compared to WT mice [225]. In addition, women with breast cancer who carried a TLR4 loss of function polymorphism (Asp299Gly) were found to have a 50% increase in the frequency of metastasis compared to women with the normal allele [225]. These findings raise concerns that inhibition of TLR4 may interfere with the chemotherapeutic and immune response to cisplatin. 

## 6. Summary

Nephrotoxicity is a serious and dose-limiting toxicity of cisplatin. Cisplatin nephrotoxicity is the composite result of the transport of cisplatin into renal epithelial cells, injury to nuclear and mitochondrial DNA, activation of a multiple cell death and survival pathways and initiation of a robust inflammatory response. Although this scheme presents many possible therapeutic targets, single interventions in animal models have generally provided only incomplete protection. Moreover, the impact of many interventions on the chemotherapeutic efficacy of cisplatin has not been adequately examined. Moving forward, combinatorial strategies which target multiple mechanisms, such as reducing cisplatin uptake and reducing inflammation, may offer the best chance for clinically meaningful prevention. Any proposed strategy, however, must be carefully studied in tumor-bearing animals to ensure that the chemotherapeutic efficacy of cisplatin is not compromised. 
